# The genome sequence of the spider-hunting wasp, *Priocnemis perturbator* (Harris, 1780) (Hymenoptera: Pompilidae)

**DOI:** 10.12688/wellcomeopenres.25074.1

**Published:** 2025-10-24

**Authors:** Liam M. Crowley, University of Oxford and Wytham Woods Genome Acquisition Lab, Darwin Tree of Life Barcoding Collective, Wellcome Sanger Institute Tree of Life Management, Samples and Laboratory team, Wellcome Sanger Institute Scientific Operations: Sequencing Operations, Wellcome Sanger Institute Tree of Life Core Informatics team, Tree of Life Core Informatics collective, Darwin Tree of Life Consortium

**Affiliations:** 1 University of Oxford, Oxford, England, UK

**Keywords:** Priocnemis perturbator; spider-hunting wasp; genome sequence; chromosomal; Hymenoptera

## Abstract

We present a genome assembly from an individual female *Priocnemis perturbator* (spider-hunting wasp; Arthropoda; Insecta; Hymenoptera; Pompilidae). The genome sequence has a total length of 391.62 megabases. Most of the assembly (67.88%) is scaffolded into 15 chromosomal pseudomolecules. The mitochondrial genome has also been assembled, with a length of 28.93 kilobases. Gene annotation of this assembly on Ensembl identified 24 581 protein-coding genes. This assembly was generated as part of the Darwin Tree of Life project, which produces reference genomes for eukaryotic species found in Britain and Ireland.

## Species taxonomy

Eukaryota; Opisthokonta; Metazoa; Eumetazoa; Bilateria; Protostomia; Ecdysozoa; Panarthropoda; Arthropoda; Mandibulata; Pancrustacea; Hexapoda; Insecta; Dicondylia; Pterygota; Neoptera; Endopterygota; Hymenoptera; Apocrita; Aculeata; Pompiloidea; Pompilidae; Pepsinae; *Priocnemis*; *Priocnemis perturbator* (Harris, 1780) (NCBI:txid1747324)

## Background

*Priocnemis perturbator* (Harris, 1780) is a large spider-hunting wasp (Pompilidae: subgenus *Umbripennis*). The family Pompilidae comprises solitary aculeate wasps that prey exclusively upon spiders. The female wasps hunt and paralyse spiders, providing the nest cell with a single paralysed spider on which the larva develops ( Edwards, 2002). Reported prey of *P. perturbator* are mainly larger ground-active species in Lycosidae and Gnaphosidae, and *Trochosa terricola* has been recorded as a prey item ( Edwards, 2002). Adults are often seen in spring in open woodland and other dry sites and visit flowers, notably wood spurge, but also blackthorn, dandelion, hawthorn and willow ( Edwards, 2002). The species flies from April to July (sometimes later) ( Edwards, 2002; O’Hanlon & O’Connor, 2021).

In Ireland it is widespread and among the most frequently recorded pompilids, and it is the largest Irish pompilid ( O’Hanlon & O’Connor, 2021). In Britain it is not regarded as scarce or threatened ( Edwards, 2002). Because identifications have historically been confused with *P. susterai*, older records should be treated cautiously, and occurrence maps based on unvetted aggregators may include misidentifications ( Edwards, 2002).

**Figure 1.**
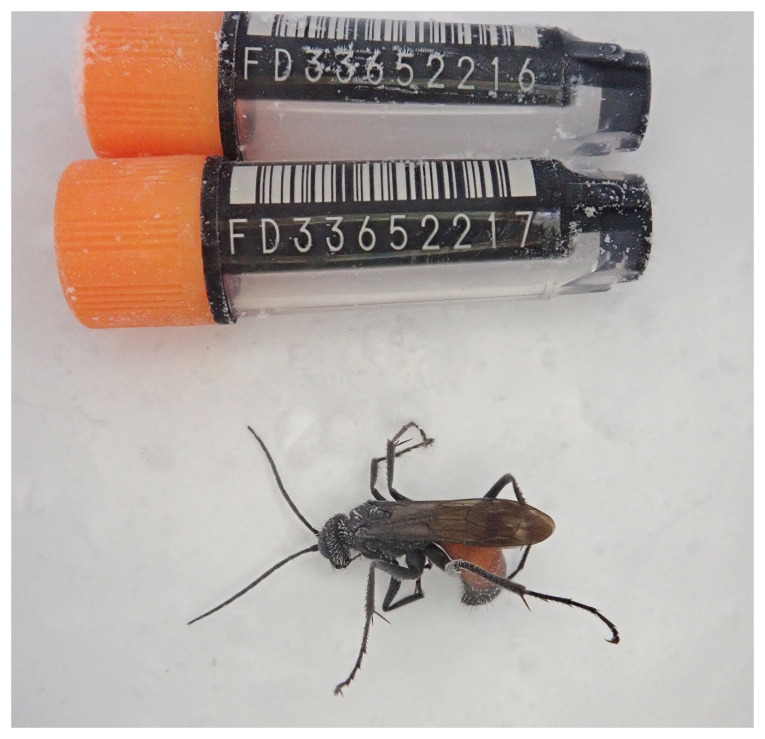
Photograph of the *Priocnemis perturbator* (iyPriPert1) specimen used for genome sequencing.

We present a chromosome-level genome sequence for the spider-hunting wasp *Priocnemis perturbator*. This assembly is the first publicly available genome for the genus *Priocnemis* and is among the few pompilid genomes currently available (confirmed via an NCBI Datasets search on 2 October 2025) ( O’Leary *et al.*, 2024). The assembly was produced using the Tree of Life pipeline using a specimen collected from Wytham Woods, Oxfordshire, UK ( Figure 1).

## Methods

### Sample acquisition and DNA barcoding

The specimen used for genome sequencing was an adult female *Priocnemis perturbator* (specimen ID Ox002176, ToLID iyPriPert1; Figure 1), collected from Wytham Woods, Oxfordshire, UK (latitude 51.769, longitude -1.328) on 2022-05-19. The specimen was collected and identified by Liam Crowley (University of Oxford). Sample metadata were collected in line with the Darwin Tree of Life project standards described by Lawniczak *et al*. (2022).

The initial identification was verified by an additional DNA barcoding process according to the framework developed by Twyford *et al.* (2024). A small sample was dissected from the specimen and stored in ethanol, while the remaining parts were shipped on dry ice to the Wellcome Sanger Institute (WSI) (see the protocol). The tissue was lysed, the COI marker region was amplified by PCR, and amplicons were sequenced and compared to the BOLD database, confirming the species identification ( Crowley *et al.*, 2023). Following whole genome sequence generation, the relevant DNA barcode region was also used alongside the initial barcoding data for sample tracking at the WSI ( Twyford *et al.*, 2024). The standard operating procedures for Darwin Tree of Life barcoding are available on protocols.io.

### Nucleic acid extraction

Protocols for high molecular weight (HMW) DNA extraction developed at the Wellcome Sanger Institute (WSI) Tree of Life Core Laboratory are available on protocols.io ( Howard *et al.*, 2025). The iyPriPert1 sample was weighed and triaged to determine the appropriate extraction protocol. Tissue from the head and thorax was homogenised by powermashing using a PowerMasher II tissue disruptor.

HMW DNA was extracted in the WSI Scientific Operations core using the Automated MagAttract v2 protocol. DNA was sheared into an average fragment size of 12–20 kb following the Megaruptor®3 for LI PacBio protocol. Sheared DNA was purified by automated SPRI (solid-phase reversible immobilisation). The concentration of the sheared and purified DNA was assessed using a Nanodrop spectrophotometer and Qubit Fluorometer using the Qubit dsDNA High Sensitivity Assay kit. Fragment size distribution was evaluated by running the sample on the FemtoPulse system. For this sample, the final post-shearing DNA had a Qubit concentration of 41.4 ng/μL and a yield of 1 656.00 ng, with a fragment size of 15.6 kb.

### PacBio HiFi library preparation and sequencing

Library preparation and sequencing were performed at the WSI Scientific Operations core. Libraries were prepared using the SMRTbell Prep Kit 3.0 (Pacific Biosciences, California, USA), following the manufacturer’s instructions. The kit includes reagents for end repair/A-tailing, adapter ligation, post-ligation SMRTbell bead clean-up, and nuclease treatment. Size selection and clean-up were performed using diluted AMPure PB beads (Pacific Biosciences). DNA concentration was quantified using a Qubit Fluorometer v4.0 (ThermoFisher Scientific) and the Qubit 1X dsDNA HS assay kit. Final library fragment size was assessed with the Agilent Femto Pulse Automated Pulsed Field CE Instrument (Agilent Technologies) using the gDNA 55 kb BAC analysis kit.

The sample was sequenced using the Sequel IIe system (Pacific Biosciences, California, USA). The concentration of the library loaded onto the Sequel IIe was in the range 40–135 pM. The SMRT link software, a PacBio web-based end-to-end workflow manager, was used to set-up and monitor the run, and to perform primary and secondary analysis of the data upon completion.

### Hi-C

***Sample preparation and crosslinking***

The Hi-C sample was prepared from 20–50 mg of frozen tissue from the head and thorax of the iyPriPert1 sample using the Arima-HiC v2 kit (Arima Genomics). Following the manufacturer’s instructions, tissue was fixed and DNA crosslinked using TC buffer to a final formaldehyde concentration of 2%. The tissue was homogenised using the Diagnocine Power Masher-II. Crosslinked DNA was digested with a restriction enzyme master mix, biotinylated, and ligated. Clean-up was performed with SPRISelect beads before library preparation. DNA concentration was measured with the Qubit Fluorometer (Thermo Fisher Scientific) and Qubit HS Assay Kit. The biotinylation percentage was estimated using the Arima-HiC v2 QC beads.

***Hi-C library preparation and sequencing***

Biotinylated DNA constructs were fragmented using a Covaris E220 sonicator and size selected to 400–600 bp using SPRISelect beads. DNA was enriched with Arima-HiC v2 kit Enrichment beads. End repair, A-tailing, and adapter ligation were carried out with the NEBNext Ultra II DNA Library Prep Kit (New England Biolabs), following a modified protocol where library preparation occurs while DNA remains bound to the Enrichment beads. Library amplification was performed using KAPA HiFi HotStart mix and a custom Unique Dual Index (UDI) barcode set (Integrated DNA Technologies). Depending on sample concentration and biotinylation percentage determined at the crosslinking stage, libraries were amplified with 10–16 PCR cycles. Post-PCR clean-up was performed with SPRISelect beads. Libraries were quantified using the AccuClear Ultra High Sensitivity dsDNA Standards Assay Kit (Biotium) and a FLUOstar Omega plate reader (BMG Labtech).

Prior to sequencing, libraries were normalised to 10 ng/μL. Normalised libraries were quantified again and equimolar and/or weighted 2.8 nM pools. Pool concentrations were checked using the Agilent 4200 TapeStation (Agilent) with High Sensitivity D500 reagents before sequencing. Sequencing was performed using paired-end 150 bp reads on the Illumina NovaSeq 6000.

### Genome assembly

Prior to assembly of the PacBio HiFi reads, a database of *k*-mer counts ( *k* = 31) was generated from the filtered reads using FastK. GenomeScope2 ( Ranallo-Benavidez *et al.*, 2020) was used to analyse the *k*-mer frequency distributions, providing estimates of genome size, heterozygosity, and repeat content.

The HiFi reads were assembled using Hifiasm ( Cheng *et al.*, 2021) with the --primary option. Haplotypic duplications were identified and removed using purge_dups ( Guan *et al.*, 2020). The Hi-C reads ( Rao *et al.*, 2014) were mapped to the primary contigs using bwa-mem2 ( Vasimuddin *et al.*, 2019), and the contigs were scaffolded in YaHS ( Zhou *et al.*, 2023) with the --break option for handling potential misassemblies. The scaffolded assemblies were evaluated using Gfastats ( Formenti *et al.*, 2022), BUSCO ( Manni *et al.*, 2021) and MERQURY.FK ( Rhie *et al.*, 2020). The organelle genomes were assembled using MitoHiFi ( Uliano-Silva *et al.*, 2023).

### Assembly curation

The assembly was decontaminated using the Assembly Screen for Cobionts and Contaminants ( ASCC) pipeline. TreeVal was used to generate the flat files and maps for use in curation. Manual curation was conducted primarily in PretextView and HiGlass ( Kerpedjiev *et al.*, 2018). Scaffolds were visually inspected and corrected as described by Howe *et al*. (2021). Manual corrections included seven joins. This reduced the scaffold count by 1.2% and increased the scaffold N50 by 4.4%. The curation process is described at https://gitlab.com/wtsi-grit/rapid-curation. PretextSnapshot was used to generate a Hi-C contact map of the final assembly.

### Assembly quality assessment

The Merqury.FK tool ( Rhie *et al.*, 2020) was run in a Singularity container ( Kurtzer *et al.*, 2017) to evaluate *k*-mer completeness and assembly quality for the primary and alternate haplotypes using the *k*-mer databases ( *k* = 31) computed prior to genome assembly. The analysis outputs included assembly QV scores and completeness statistics.

The genome was analysed using the BlobToolKit pipeline, a Nextflow implementation of the earlier Snakemake version ( Challis *et al.*, 2020). The pipeline aligns PacBio reads using minimap2 ( Li, 2018) and SAMtools ( Danecek *et al.*, 2021) to generate coverage tracks. It runs BUSCO ( Manni *et al.*, 2021) using lineages identified from the NCBI Taxonomy ( Schoch *et al.*, 2020). For the three domain-level lineages, BUSCO genes are aligned to the UniProt Reference Proteomes database ( Bateman *et al.*, 2023) using DIAMOND blastp ( Buchfink *et al.*, 2021). The genome is divided into chunks based on the density of BUSCO genes from the closest taxonomic lineage, and each chunk is aligned to the UniProt Reference Proteomes database with DIAMOND blastx. Sequences without hits are chunked using seqtk and aligned to the NT database with blastn ( Altschul *et al.*, 1990). The BlobToolKit suite consolidates all outputs into a blobdir for visualisation. The BlobToolKit pipeline was developed using nf-core tooling ( Ewels *et al.*, 2020) and MultiQC ( Ewels *et al.*, 2016), with containerisation through Docker ( Merkel, 2014) and Singularity ( Kurtzer *et al.*, 2017).

## Genome sequence report

### Sequence data

**Figure 2.**
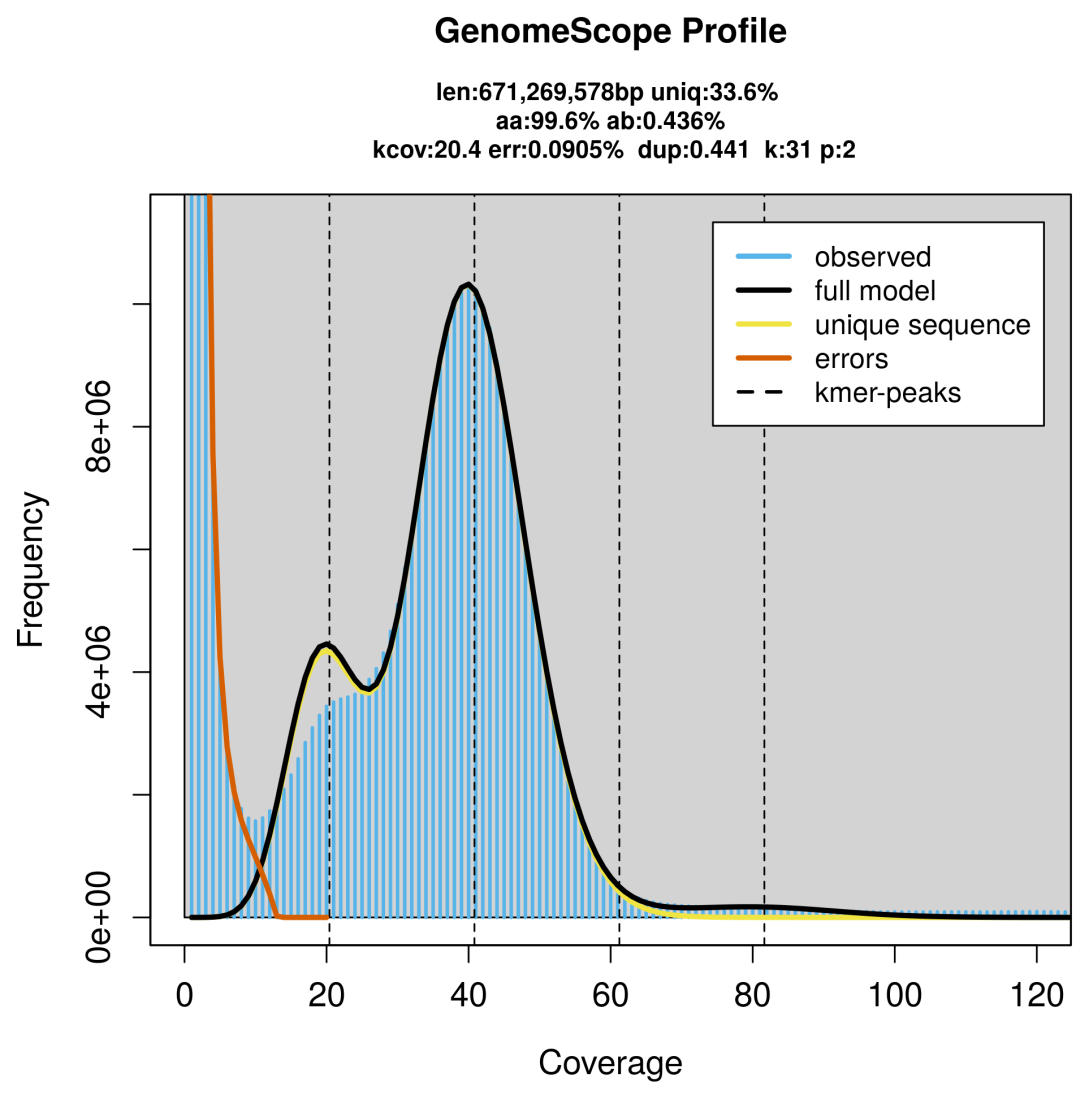
Frequency distribution of *k*-mers generated using GenomeScope2. The plot shows observed and modelled *k*-mer spectra, providing estimates of genome size, heterozygosity, and repeat content based on unassembled sequencing reads.

PacBio sequencing of the *Priocnemis perturbator* specimen generated 28.25 Gb (gigabases) from 2.24 million reads, which were used to assemble the genome. GenomeScope2.0 analysis estimated the haploid genome size at 671.27 Mb, with a heterozygosity of 0.44% and repeat content of 66.52% ( Figure 2). These estimates guided expectations for the assembly. Based on the estimated genome size, the sequencing data provided approximately 41× coverage. Hi-C sequencing produced 115.54 Gb from 765.18 million reads, which were used to scaffold the assembly. Table 1 summarises the specimen and sequencing details.

**Table 1.** Specimen and sequencing data for BioProject PRJEB61485.

Platform	PacBio HiFi	Hi-C
**ToLID**	iyPriPert1	iyPriPert1
**Specimen ID**	Ox002176	Ox002176
**BioSample (source** ** individual)**	SAMEA110451618	SAMEA110451618
**BioSample (tissue)**	SAMEA110451826	SAMEA110451826
**Tissue**	head and thorax	head and thorax
**Instrument**	Sequel IIe	Illumina NovaSeq 6000
**Run accessions**	ERR11263493	ERR11271511
**Read count total**	2.24 million	765.18 million
**Base count total**	28.25 Gb	115.54 Gb

### Assembly statistics

The sequenced specimen is a diploid female wasp. The primary haplotype was assembled, and contigs corresponding to an alternate haplotype were also deposited in INSDC databases. The final assembly has a total length of 391.62 Mb in 479 scaffolds, with 137 gaps, and a scaffold N50 of 16.13 Mb ( Table 2).

**Table 2.** Genome assembly statistics.

**Assembly name**	iyPriPert1.1
**Assembly accession**	GCA_963942575.1
**Alternate haplotype accession**	GCA_963942535.1
**Assembly level**	chromosome
**Span (Mb)**	391.62
**Number of chromosomes**	15
**Number of contigs**	616
**Contig N50**	2.39 Mb
**Number of scaffolds**	479
**Scaffold N50**	16.13 Mb
**Organelles**	Mitochondrion: 28.93 kb

**Figure 3.**
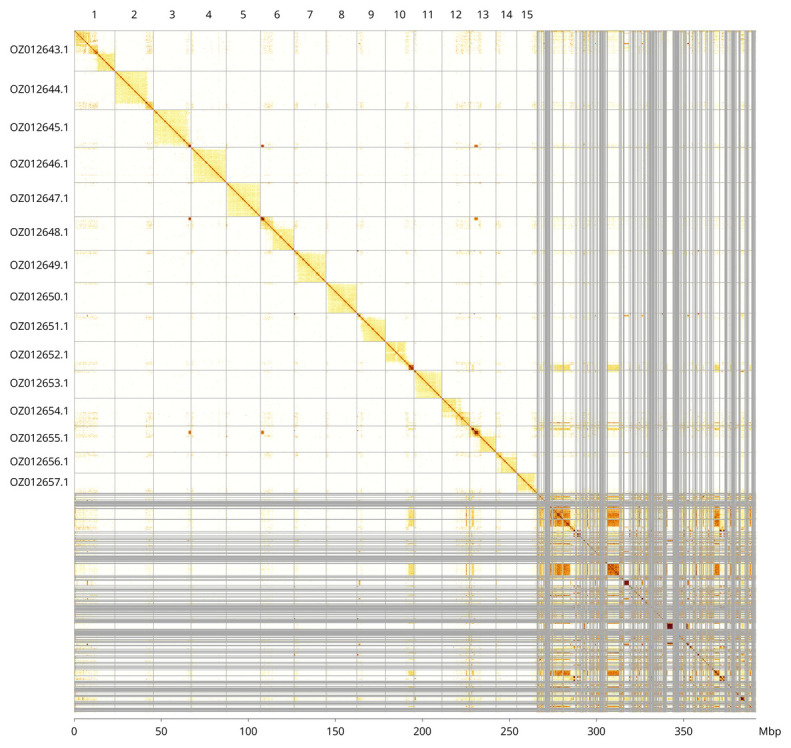
Hi-C contact map of the *Priocnemis perturbator* genome assembly. Assembled chromosomes are shown in order of size and labelled along the axes, with a megabase scale shown below. The plot was generated using PretextSnapshot.

The assembly sequence was assigned to 15 chromosomal-level scaffolds. These chromosome-level scaffolds, confirmed by Hi-C data, are named according to size ( Figure 3; Table 3). Many short, repeat-rich scaffolds lack informative Hi-C contacts and remain unlocalised, which is common in hymenopteran assemblies.

**Table 3.** Chromosomal pseudomolecules in the primary genome assembly of *Priocnemis perturbator* iyPriPert1.

INSDC accession	Molecule	Length (Mb)	GC%
OZ012643.1	1	23.40	45
OZ012644.1	2	22.21	46.50
OZ012645.1	3	21.56	46.50
OZ012646.1	4	20.18	46
OZ012647.1	5	19.59	46.50
OZ012648.1	6	19.35	45.50
OZ012649.1	7	18.43	46
OZ012650.1	8	17.71	47
OZ012651.1	9	16.39	44.50
OZ012652.1	10	16.30	46
OZ012653.1	11	16.13	46
OZ012654.1	12	15.97	43.50
OZ012655.1	13	15.01	43.50
OZ012656.1	14	12.07	45
OZ012657.1	15	11.54	45.50

The mitochondrial genome was also assembled (length 28.93 kb, OZ012658.1). This sequence is included as a contig in the multifasta file of the genome submission and as a standalone record.

**Figure 4.**
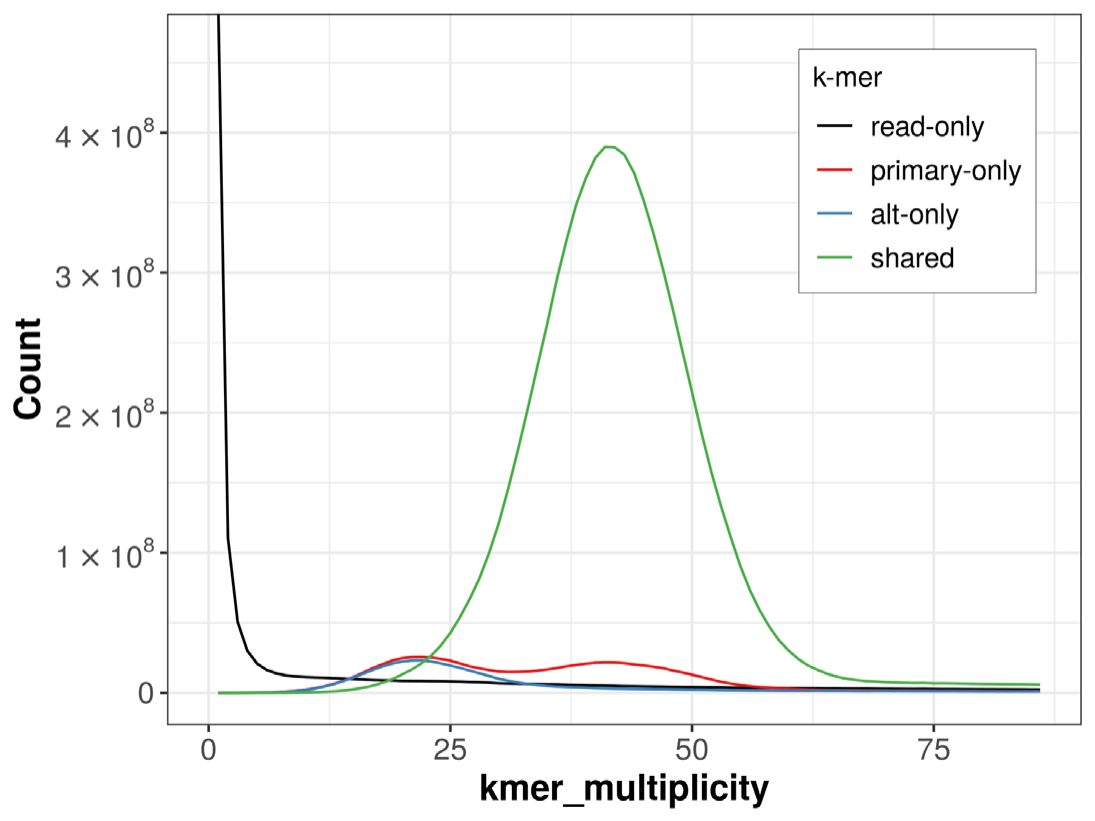
Evaluation of *k*-mer completeness using MerquryFK. This plot illustrates the recovery of *k*-mers from the original read data in the final assemblies. The horizontal axis represents *k*-mer multiplicity, and the vertical axis shows the number of *k*-mers. The black curve represents *k*-mers that appear in the reads but are not assembled. The green curve corresponds to *k*-mers shared by both haplotypes, and the red and blue curves show *k*-mers found only in one of the haplotypes.

The combined primary and alternate assemblies achieve an estimated QV of 62.2. The *k*-mer completeness is 86.42% for the primary assembly, 82.70% for the alternate haplotype, and 93.56% for the combined assemblies ( Figure 4).

**Figure 5.**
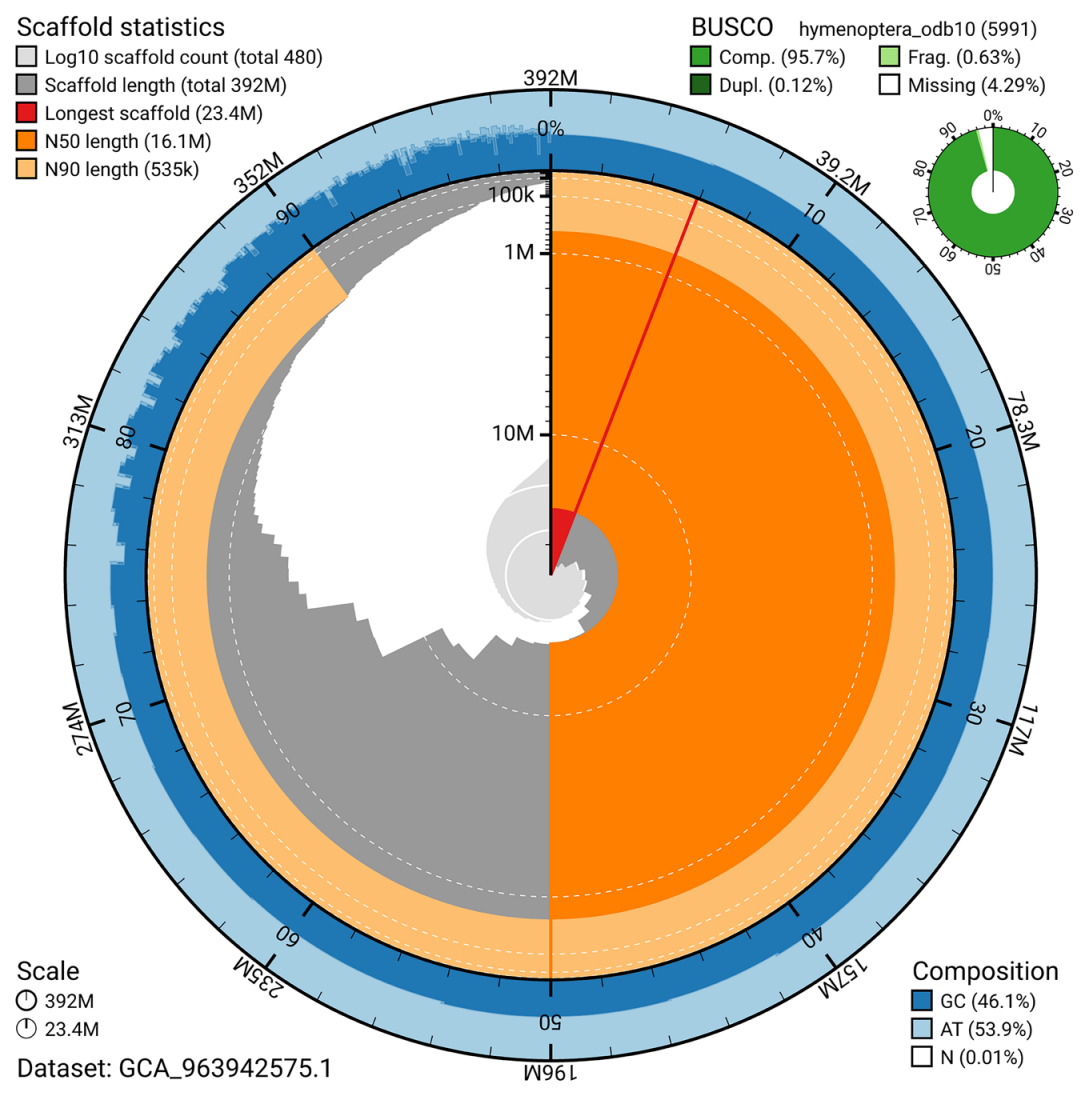
Assembly metrics for iyPriPert1.1. The BlobToolKit snail plot provides an overview of assembly metrics and BUSCO gene completeness. The circumference represents the length of the whole genome sequence, and the main plot is divided into 1 000 bins around the circumference. The outermost blue tracks display the distribution of GC, AT, and N percentages across the bins. Scaffolds are arranged clockwise from longest to shortest and are depicted in dark grey. The longest scaffold is indicated by the red arc, and the deeper orange and pale orange arcs represent the N50 and N90 lengths. A light grey spiral at the centre shows the cumulative scaffold count on a logarithmic scale. A summary of complete, fragmented, duplicated, and missing BUSCO genes in the hymenoptera_odb10 set is presented at the top right. An interactive version of this figure can be accessed on the BlobToolKit viewer.

**Figure 6.**
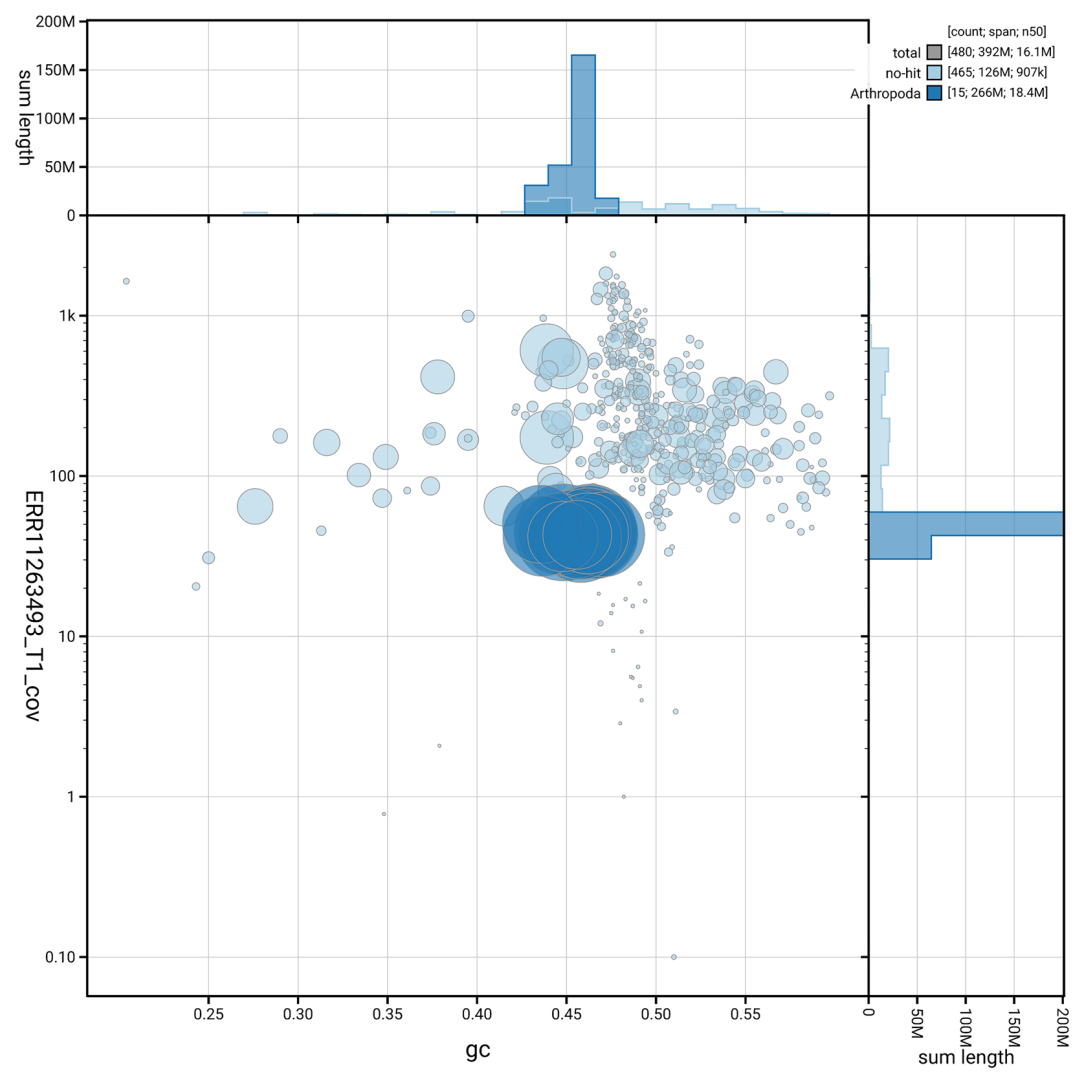
BlobToolKit GC-coverage plot for iyPriPert1.1. Blob plot showing sequence coverage (vertical axis) and GC content (horizontal axis). The circles represent scaffolds, with the size proportional to scaffold length and the colour representing phylum membership. The histograms along the axes display the total length of sequences distributed across different levels of coverage and GC content. An interactive version of this figure is available on the BlobToolKit viewer.

BUSCO v.5.5.0 analysis using the hymenoptera_odb10 reference set ( *n* = 5 991) identified 95.7% of the expected gene set (single = 95.6%, duplicated = 0.1%). The snail plot in Figure 5 summarises the scaffold length distribution and other assembly statistics for the primary assembly. The blob plot in Figure 6 shows the distribution of scaffolds by GC proportion and coverage.

Table 4 lists the assembly metric benchmarks adapted from Rhie *et al.* (2021) and the Earth BioGenome Project Report on Assembly Standards September 2024. The EBP metric, calculated for the primary assembly, is **6.7.Q61**.

**Table 4.** Earth Biogenome Project summary metrics for the *Priocnemis perturbator* assembly.

Measure	Value	Benchmark
EBP summary (primary)	6.7.Q61	6.C.Q40
Contig N50 length	2.39 Mb	≥ 1 Mb
Scaffold N50 length	16.13 Mb	= chromosome N50
Consensus quality (QV)	Primary: 61.8; alternate: 62.8; combined: 62.2	≥ 40
*k*-mer completeness	Primary: 86.42%; alternate: 82.70%; combined: 93.56%	≥ 95%
BUSCO	C:95.7% [S:95.6%; D:0.1%]; F:0.6%; M:3.7%; n:5 991	S > 90%; D < 5%
Percentage of assembly assigned to chromosomes	67.88%	≥ 90%

## Genome annotation report

The *Priocnemis perturbator* genome assembly (GCA_963942575.1) was annotated by Ensembl at the European Bioinformatics Institute (EBI). This annotation includes 24 917 transcribed mRNAs from 24 581 protein-coding genes. The average transcript length is 2 752.14 bp, with an average of 3.84 exons per transcript. For further information about the annotation, please refer to the Ensembl annotation page.

### Wellcome Sanger Institute – Legal and Governance

The materials that have contributed to this genome note have been supplied by a Darwin Tree of Life Partner. The submission of materials by a Darwin Tree of Life Partner is subject to the **‘Darwin Tree of Life Project Sampling Code of Practice’**, which can be found in full on the Darwin Tree of Life website. By agreeing with and signing up to the Sampling Code of Practice, the Darwin Tree of Life Partner agrees they will meet the legal and ethical requirements and standards set out within this document in respect of all samples acquired for, and supplied to, the Darwin Tree of Life Project. Further, the Wellcome Sanger Institute employs a process whereby due diligence is carried out proportionate to the nature of the materials themselves, and the circumstances under which they have been/are to be collected and provided for use. The purpose of this is to address and mitigate any potential legal and/or ethical implications of receipt and use of the materials as part of the research project, and to ensure that in doing so we align with best practice wherever possible. The overarching areas of consideration are:

- Ethical review of provenance and sourcing of the material
- Legality of collection, transfer and use (national and international)

Each transfer of samples is further undertaken according to a Research Collaboration Agreement or Material Transfer Agreement entered into by the Darwin Tree of Life Partner, Genome Research Limited (operating as the Wellcome Sanger Institute), and in some circumstances, other Darwin Tree of Life collaborators.

## Data Availability

European Nucleotide Archive: Priocnemis perturbator. Accession number PRJEB61485. The genome sequence is released openly for reuse. The *Priocnemis perturbator* genome sequencing initiative is part of the Darwin Tree of Life Project (PRJEB40665) and the Sanger Institute Tree of Life Programme (PRJEB43745). All raw sequence data and the assembly have been deposited in INSDC databases. Raw data and assembly accession identifiers are reported in Table 1 and Table 2. Production code used in genome assembly at the WSI Tree of Life is available at https://github.com/sanger-tol. Table 5 lists software versions used in this study.

## Author information

Contributors are listed at the following links:

- Members of the University of Oxford and Wytham Woods Genome Acquisition Lab
- Members of the Darwin Tree of Life Barcoding collective
- Members of the Wellcome Sanger Institute Tree of Life Management, Samples and Laboratory team
- Members of Wellcome Sanger Institute Scientific Operations – Sequencing Operations
- Members of the Wellcome Sanger Institute Tree of Life Core Informatics team
- Members of the Tree of Life Core Informatics collective
- Members of the Darwin Tree of Life Consortium
