# Supplementary material for: The genome sequence of the spider-hunting wasp, Priocnemis perturbator (Harris, 1780) (Hymenoptera: Pompilidae)
Source: Wellcome Open Res. 2025 Oct 24;10:599. [Version 1] doi: 10.12688/wellcomeopenres.25074.1 (PMC12770895; doi:10.12688/wellcomeopenres.25074.1)
Supplement: Peer review history

# Reviewer report: Reviewer response for version 1

**DOI:** 10.21956/wellcomeopenres.27641.r138459

**Published:** 2026-01-05

**Authors:** Julien Varaldi, Sara Oukkal

**Affiliations:** 1 Universite Claude Bernard Lyon, Villeurbanne, Auvergne-Rhône-Alpes, France; 2 UMR CNRS 5558 - LBBE, Universite Claude Bernard Lyon 1, Villeurbanne, Auvergne-Rhône-Alpes, France

This paper presents a full genome sequence for the spider hunting wasp, Priocnemis perturbator (Pompilidae). This is useful, given that very few genomic data are available for this family. In the introduction, the authors provide an overview of the biology of the wasp, including its lifecycle and geographic distribution. Starting from a single diploid female, the authors produced an assembly (and an alternative haplotype) using PacBio HiFi and HiC data. The resulting assembly comprises 15 putative chromosomes. The methods are sound and the resulting assembly is valuable.

 The main criticism is that only 68% of the PacBio assembly is included in the final assembly which is rather low and necessitates explanation. Based on the BlobToolKit plot, it is seems that many of these sequences actually belong to the wasp's associated microbiota. Some discussion of this would have been useful.

Are sufficient details of methods and materials provided to allow replication by others?

Yes

Is the rationale for creating the dataset(s) clearly described?

Yes

Are the datasets clearly presented in a useable and accessible format?

Yes

Are the protocols appropriate and is the work technically sound?

Yes

Reviewer Expertise:

evolutionary genomics

We confirm that we have read this submission and believe that we have an appropriate level of expertise to confirm that it is of an acceptable scientific standard.

# Reviewer report: Reviewer response for version 1

**DOI:** 10.21956/wellcomeopenres.27641.r141196

**Published:** 2025-12-24

**Authors:** Andrew J Severin

**Affiliations:** 1 Iowa State University, Ames, Iowa, USA

This genome note describes the genome assembly and annotation of the spider-hunting was ( *Priocnemis perturbator).  *Results of the assembly indicate a chromosomal level assembly with many unplaced highly repetitive scaffolds with 95%+ completeness based on BUSCO. Software versions were included along with a github organization repository for easier replication of results.  Data appears to be present on ENA browser.

 This genome will be a great resource to researchers studying wasps expecially in the genus Priocnemis.

Are sufficient details of methods and materials provided to allow replication by others?

Yes

Is the rationale for creating the dataset(s) clearly described?

Yes

Are the datasets clearly presented in a useable and accessible format?

Yes

Are the protocols appropriate and is the work technically sound?

Yes

Reviewer Expertise:

Bioinformatics, genome assembly and annotation.

I confirm that I have read this submission and believe that I have an appropriate level of expertise to confirm that it is of an acceptable scientific standard.
